# Can Risk Factors and Opportunities to Be Observed Explain Why Culturally and Linguistically Diverse Children Have Less Child Protection Contact?

**DOI:** 10.1111/jpc.70036

**Published:** 2025-03-20

**Authors:** Razlyn Abdul Rahim, R. Pilkington, K. D'Onise, J. Lynch

**Affiliations:** ^1^ School of Public Health, The University of Adelaide Adelaide South Australia Australia; ^2^ Robinson Research Institute, The University of Adelaide Adelaide South Australia Australia; ^3^ Bristol Medical School, Population Health Sciences, University of Bristol Bristol UK

**Keywords:** child abuse, culturally and linguistically diverse, healthcare disparities, socioeconomic factors

## Abstract

**Aim:**

Compared prevalence of risk factors for child protection (CP) contact and contact patterns with health, education and housing systems as opportunities to be observed for reporting to CP between Culturally and Linguistically Diverse (CALD) and non‐CALD children.

**Method:**

Health, births, education and public housing data for children and parents from 12 months before the child's birth to age 7 from the South Australian Better Evidence, Better Outcomes, Linked Data platform. Participants: SA‐born children in their first year in public school from 2009 to 2015 (*n* = 76 563). CALD: non‐Indigenous, language other than English/Indigenous/Sign, or at least one parent born in a non‐English speaking country. Outcomes: antenatal visits, 1–4‐week check attendance, emergency presentations, and hospital admissions (0–7 years), preschool attendance, parental records for mental health, alcohol and other drug (AOD) use, self‐harm, family domestic violence (FDV), maltreatment and housing insufficiency.

**Results:**

Contact for antenatal visits, 1–4‐week check, and hospital admissions (0–7 years) were comparable across both groups. CALD children had more emergency presentations (RD 7.7% points, 95% CI 6.8–8.9). By age 7, more non‐CALD children had at least one parent with mental health issues (RD 5.9 [95% CI 5.3–6.6]), AOD (RD 5.8 [95% CI 5.4–6.2]) and housing insufficiency (RD 7.8 [95% CI 6.9–8.6]). The prevalence of other risk factors was similar across both groups.

**Conclusion:**

The lower CP contact in CALD children is likely explained by a lower prevalence of CP risk factors and not due to fewer opportunities to be observed in their contact with the three systems.

**Summary**

- What is already known on this topic
  ○ CALD children have lower CP contact that non‐CALD children at all levels of contact from initial reporting to out‐of‐home care [1].
- What this paper adds
  ○ CALD children have lower background risk for CP contact compared to non‐CALD children.
  ○ CALD and non‐CALD children have equal opportunities to be observed for CP contact through their contact with the health, education and public housing systems.
  ○ The lower CP contact observed in CALD children is likely explained by lower background risk for CP contact and not due to fewer opportunities to be observed by mandatory reporters in the health, education and public housing systems, compared to non‐CALD children.

## 1 Introduction

Child maltreatment is an important public health issue with adverse repercussions for children's mental and physical health, and their social and economic capabilities in later life [2, 3, 4, 5, 6, 7]. A public health approach to child maltreatment begins with early support for at‐risk populations through addressing underlying factors that drive contact with child protection (CP) agencies. In this study we use contact with the CP system as a proxy for child maltreatment. We have previously shown that in South Australia (SA), by age 7, children of Culturally and Linguistically Diverse (CALD) background experience lower CP contact across all levels from initial reporting to out‐of‐home care [1]. Similar observations related to ethnic diversity have been observed in the USA [8, 9], Canada [10] and United Kingdom [11, 12]. Although we were unable to empirically test this in the prior study, one of our hypotheses for lower CP contact for CALD children is that they have less contact with agencies staffed by mandated reporters to CP, such as the health, education and housing systems. In the UK context, Bywaters and colleagues proposed a framework consisting of three elements to understand ethnic differences in CP contact [13]. These were described as ‘artefactual issues’ related to ‘data quality and coverage’; ‘supply factors’ related to activities of surveillance and mandatory reporting from groups such as healthcare workers and educators; and ‘demand factors’ related to risk factors or conditions that contribute to occurrence of child maltreatment [13].

We showed lower levels of CP contact in CALD children were unlikely to be explained by ‘artefactual issues’ related to data quality [13] because findings were consistent when different definitions of CALD were used from data sources outside of the CP system [1]. Furthermore, socioeconomic characteristics at birth and receipt of the low‐income school card at school entry were similar between groups, suggesting that aspects of ‘demand factors’ [13] could not explain the lower CP contact [1]. The current study has two objectives; (i) to compare ‘supply factors’ related to opportunities to be observed for CP surveillance by mandated reporters through health system encounters; and (ii) compare ‘demand factors’ related to the distribution of risk factors for being reported to CP, such as parental mental health issues, drug and alcohol abuse, family and domestic violence and housing insufficiency, for CALD and non‐CALD children. The analysis is deliberately descriptive and consistent with recently recommended best practice [14, 15].

## 2 Method

### 2.1 Data Sources

This study used whole population de‐identified data from the SA Better Evidence, Better Outcomes Linked Data (BEBOLD) platform. BEBOLD contains linked administrative data from the Perinatal Statistics Collection, Births, Deaths and Marriages Registry, Government School Enrolment Census (SEC), the 2009, 2012 and 2015 Australian Early Development Census (AEDC), public hospital inpatients (ISAAC), Emergency Department presentations (EDDC), Child and Family Health Service (CAFHS) child health and development checks and SA public housing waitlist, tenancies and private rental assessments, assistance and occupants records. The BEBOLD platform also contains ‘family files’ based on birth registrations where de‐identified mother and co‐parent information is linked to children, and so are only available for children whose birth is registered in SA.

Linkage was conducted by SANT DataLink, an independent agency [16]. The estimated false positive linkage error is 0.4% [17].

### 2.2 Study Population

**FIGURE 1:**
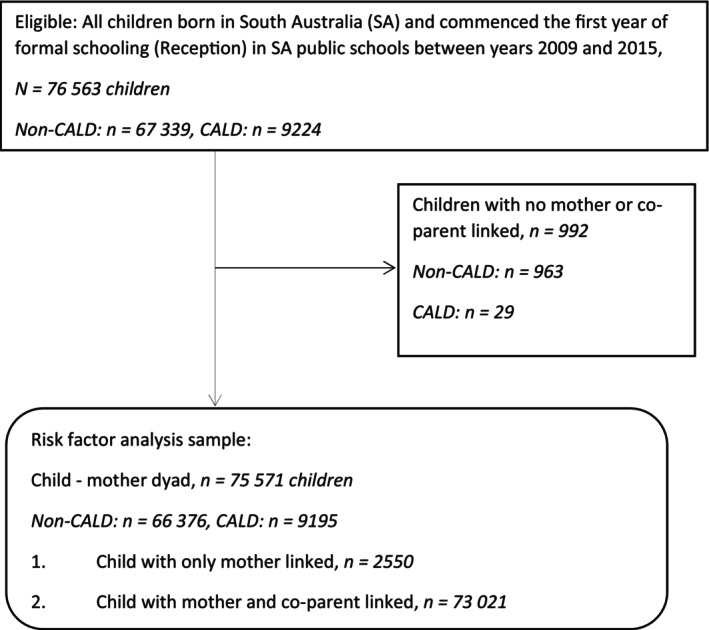
Study population for main analysis.

For the main analysis (Figure 1) assessing the opportunity to be observed in CP, the study population included children born in SA who started in a government school between 2009 and 2015 (*N* = 76 563), corresponding to children born 2003–2010. A subset of this population (*n* = 75 571) had at least one parent linked to their record and was included in the main analysis to assess the distribution of risk factors for child maltreatment from birth to age 7. Age 7 reflects the maximum follow‐up period across all reception cohorts available at the time of analysis. The study population comprised of government school attendees as the information used to best ascertain CALD is obtained from the SEC available for government schools only on the BEBOLD platform.

For sensitivity analysis (Figure S1) we assessed the opportunity to be observed among children who sat the AEDC in the years 2009, 2012, and 2015 in SA. This population includes government, independent and Catholic schools (*N* = 46 577). Of this population, 46 104 children were linked to at least one parent and were included in examining the distribution of child maltreatment risk factors. The sensitivity analysis aimed to assess the robustness of findings with the inclusion of children from independent and Catholic schools, whose family characteristics may be different from those attending government schools.

CALD was defined as a non‐Aboriginal or Torres Strait Islander who spoke a first or second language other than English, Celtic, Australian Indigenous or sign at home, and/or had at least one parent born in countries other than Australia, New Zealand, UK, Ireland, USA, and Canada. Non‐CALD were all Aboriginal or Torres Strait Islander and non‐Indigenous peoples who spoke English, Celtic, any Australian Indigenous or sign language at home and had both parents born in Australia, New Zealand, UK, Ireland, USA, Canada or South Africa [18]. Language spoken was used to distinguish between CALD and non‐CALD children, whose parents were born in South Africa. Children who spoke English or Celtic as a first or second language were considered part of the British diaspora and therefore non‐CALD. These items were only available in the SEC for children attending government schools. See Supporting Information for the CALD definition used in the sensitivity analysis.

### 2.3 ‘Supply Factors’—Opportunities to Be Observed

Each encounter with the health system represents an opportunity for health care workers to observe the child and report concerns to CP services. We included frequency of maternal antenatal care visits (< 7, 7 to 10, > 10), attendance at the 1‐ to 4‐week child health and development check during the neonatal period conducted by CAFHS, EDDC (all diagnoses) and hospital admissions (all diagnoses) comparing CALD and non‐CALD children. EDDC and inpatient admission episodes were measured cumulatively from children's birth to ages 1, 4 and 7 years.

### 2.4 ‘Demand Factors’—Background Risk Factors

Risk factors for CP contact included parental mental health issues, alcohol and other drug use, intentional self‐harm, family and domestic violence, maltreatment and housing insufficiency. Except for housing insufficiency, risk factor information was obtained from documentation across all diagnosis fields in parental emergency department encounter records and hospital inpatient episodes based on the International Statistical Classification of Diseases and Related Health Problems, Eleventh Edition (ICD‐10‐AM) [19]. Additional information on mental health issues and family and domestic violence was obtained from public housing data. Housing insufficiency was defined by material hardship (e.g., unable to afford rent, no permanent place to stay, eviction notice, current lease expired, homeless) [20, 21] and hazardous living conditions (e.g., overcrowding, house is unsafe or in unhealthy condition, property repair/upgrade transfer, safety at risk) [22, 23]. Although hazardous living conditions usually refer to structural conditions of the dwelling, we have included overcrowding due to the increased risk of communicable diseases, mental health issues, sleep disturbances and exposure to second‐hand smoking [24]. Information on housing insufficiency was obtained from SA public housing records.

All risk factors were measured from 12 months prior to the birth of the child, and cumulatively from the child's birth to ages 1, 4 and 7 years.

## 3 Analysis

### 3.1 Main Analysis

We calculated risk differences (RD) in percentage points comparing non‐CALD and CALD children for the number of antenatal care visits mothers attended, completion of 1 to 4‐week health and development checks, ever presented to the emergency department, and ever had hospital admissions by age 7 years, and whether the child participated in non‐parental care or early childhood programmes prior to commencing formal schooling. The number of children with at least one parent with a history of mental health issues, alcohol and other drug use, intentional self‐harm, family and domestic violence, maltreatment and housing insufficiency was compared between both groups at 12 months prior to the child's birth, and cumulatively from birth to ages 1, 4 and 7 years.

### 3.2 Sensitivity Analysis

The analysis above was repeated with a study population that included all children born in SA who participated in the AEDC in all SA school sectors in the years 2009, 2012 and 2015. This necessitated changing the CALD definition used in the main analysis but allowed an assessment of the robustness of the main findings if children attending independent and Catholic schools were included.

## 4 Ethics Statement

Ethics approval was granted by the Human Research Ethics Committee of the South Australian Department of Health and Ageing (SA Health) (ref HREC/13/SAH/106; HREC/13/SAH/106/AM18), the University of Adelaide Human Research Ethics Committee (H‐185‐2011), and the Aboriginal Health Council Research Ethics Committee (ref 04‐13‐538).

## 5 Results

### 5.1 Main Analysis

Table 1 shows that the frequency of contact with the health system for non‐CALD and CALD children was largely comparable. The proportion of children whose mothers attended at least seven antenatal care visits was 85.9% for non‐CALD and 84.8% for CALD. The proportion of mothers of CALD and non‐CALD children who attended less than seven antenatal visits was comparable. Over 81% of both groups completed the 1‐ to 4‐week health check with CAFHS. CALD children had more emergency department visits than non‐CALD by age 7, representing a greater opportunity to be observed by health workers by 7.7 percentage points (95% CI 6.8–8.9). The proportion of children admitted to hospital was comparable. The proportion of children in non‐parental care or an early childhood program prior to reception was higher among non‐CALD children by 2.2 (95% CI 1.5–3.0) percentage points.

**TABLE 1:** Health care and early childhood education system contact for children born in SA who attended reception in government schools between years 2009 and 2015, followed up to age 7 years.

	Non‐CALD (*n* = 67 339)		CALD (*n* = 9224)		Total (*N* = 76 563)		Risk difference (non‐CALD − CALD)	
	*n*	col %	*n*	col %	*N*	col %	Percentage points	95% CI
Antenatal care visits mother attended								
< 7	5384	8.0	774	8.4	6158	8.0	−0.4	−1.0‐0.2
7 to 10	25 750	38.2	4285	46.5	30 035	39.2	−8.2	−9.4‐7.2
> 10	32 090	47.7	3540	38.4	35 630	46.5	9.3	8.2–10.4
Missing	4115	6.1	625	6.8	4740	6.2	−0.7	−1.2‐0.16
Completed health and development check								
1–4 week	54 964	81.6	7543	81.8	62 507	81.6	−0.2	−1.4‐0.6
By age 7 years								
Ever presented to ED	48 452	72.0	7350	79.7	55 802	72.9	−7.7	−8.9‐6.8
Ever admitted	38 027	56.5	4982	54.0	43 009	56.2	2.5	1.4–3.6
Non‐parental care/early childhood programs†								
Yes	23 437	91.8	3169	89.6	26 606	91.6	2.2	1.5–3.0
No	424	1.7	56	1.6	480	1.7	0.1	−0.2‐0.4
Don't know	1440	5.6	293	8.3	1733	6.0	−2.6	−0.03‐0.2
Missing	219	0.9	19	0.5	238	0.8	0.4	0.2–0.6

† Children who sat the AEDC in years 2009, 2012, and 2015 (*N* = 29 057, non‐CALD: *n* = 25 520, CALD: *n* = 3537).

Table 2 shows a higher proportion of non‐CALD children had at least one parent with a history of mental health issues, alcohol and other drug use and housing insufficiency from 12 months prior to the child's birth up to age 7 years. RD by age 7 were 5.9 (95% CI 5.3–6.6), 5.8 (95% CI 5.4–6.2) and 7.8 (95% CI 6.9–8.6) percentage points respectively. Both groups had similar proportions of children with at least one parent with records of intentional self‐harm, family and domestic violence and maltreatment by age 7 years with RD of 1.5 (95% CI 1.3–1.8), 1.1 (95% CI 0.7–1.5) and 0.2 (95% CI 0.1–0.3) percentage points respectively.

**TABLE 2:** Distribution of child maltreatment risk factors for children born in SA and attended reception in government schools between the years 2009 and 2015, followed from 12 months prior to birth up to age 7 years.

	Non‐CALD (*n* = 66 376)		CALD (*n* = 9195)		Total (*N* = 75 571)†		Risk difference (non‐CALD − CALD)	
	*n*	%	*n*	%	*n*	%	Percentage points	95% CI
Parental mental health issues								
12 months prior to child's birth	773	1.2	63	0.7	836	1.1	0.5	0.3–0.7
From child's birth up to age 1 year	1807	2.7	155	1.7	1964	2.6	1.0	0.7–1.3
From child's birth up to age 4 years	7032	10.6	590	6.4	7624	10.1	4.2	3.6–4.7
From child's birth up to age 7 years	10 049	15.1	847	9.2	10 896	14.4	5.9	5.3–6.6
Parental alcohol and other drug issues								
12 months prior to child's birth	181	0.3	12	0.1	193	0.3	0.1	0.1–0.2
From child's birth up to age 1 year	950	1.4	41	0.5	991	1.3	1.0	0.8–1.1
From child's birth up to age 4 years	4140	6.2	191	2.1	4331	5.7	4.2	3.8–4.5
From child's birth up to age 7 years	5882	8.9	284	3.1	6166	8.2	5.8	5.4–6.2
Parental history of intentional self‐harm								
12 months prior to child's birth	15	0.0	< 5	‡	§	0.0	0.0	0.0–0.0
From child's birth up to age 1 year	217	0.3	23	0.3	240	0.3	0.1	0.0–0.2
From child's birth up to age 4 years	1120	1.7	80	0.9	1200	1.6	0.8	0.6–1.0
From child's birth up to age 7 years	1754	2.6	104	1.1	1858	2.5	1.5	1.3–1.8
Parental family and domestic violence issues								
12 months prior to child's birth	470	0.7	35	0.4	505	0.7	0.3	0.2–0.5
From child's birth up to age 1 year	713	1.1	58	0.6	771	1.0	0.4	0.3–0.6
From child's birth up to age 4 years	2140	3.2	223	2.4	2363	3.1	0.8	0.5–1.1
From child's birth up to age 7 years	2760	4.2	280	3.1	3040	4.0	1.1	0.7–1.5
Parental maltreatment issues								
12 months prior to child's birth	44	0.1	6	0.1	50	0.1	0.0	0.0–0.0
From child's birth up to age 1 year	42	0.1	< 5	‡	§	0.1	0.1	0.0–0.1
From child's birth up to age 4 years	158	0.2	12	0.1	170	0.2	0.1	0.0–0.2
From child's birth up to age 7 years	215	0.3	15	0.2	230	0.3	0.2	0.1–0.3
Parental housing insufficiency								
12 months prior to child's birth	3098	4.7	426	4.6	3524	4.7	0.0	0.4–0.5
From child's birth up to age 1 year	5001	7.5	464	5.1	5465	7.2	2.5	2.0–3.0
From child's birth up to age 4 years	13 816	20.8	1336	14.5	15 152	20.1	6.3	5.5–7.1
From child's birth up to age 7 years	16 969	25.6	1638	17.8	18 607	24.6	7.8	6.9–8.6

† Children born in SA linked with at least one parent.

‡ Less than 0%.

§ Redacted due to the presence of cells within the same row with counts of less than five.

### 5.2 Sensitivity Analysis

Findings were consistent with the main analysis for health and early childhood education system contact, as well as risk factor distribution for non‐CALD and CALD children from Reception cohorts in years 2009, 2012 and 2015 in all SA school sectors. (Tables S1 and S2).

## 6 Discussion

This study examined whether theorised ‘supply’ and ‘demand’ factors [13] could explain why children from CALD backgrounds have less contact with CP at all levels of the system from initial contact to OOHC [1]. The health sector and police are the largest sources of notification to CP in children under five, with the health sector accounting for 20% of notifications in this group [1]. In children aged 5 to 7 years, health accounts for 11% of notifications to CP [1]. The current study showed that, up to age 7, non‐CALD and CALD children were generally comparable in the opportunities for CP surveillance by the public hospital system and CAFHS. Lack of engagement in antenatal care may result in unborn child notification to CP, particularly when the mother concurrently experiences psychosocial risks [25]. However, we showed that the proportion of mothers with inadequate antenatal care visits (less than seven) was comparable between groups. Similarly, the proportion of children known to have attended childcare services prior to formal schooling was comparable. The available data suggest that CALD children are no less likely to be observed than non‐CALD children by mandatory CP notifiers such as health care workers and educators. However, data on visits to general practice, other primary health care services and specialist outpatient visits were not available for this study. But we acknowledge that these are important areas in the health system where CP reporting may occur.

The discrepancies in the distribution of CP risk factors may explain the higher CP contact in non‐CALD compared to CALD children [1]. Again, limitations to current data availability meant that there are other data sources that may contribute towards a more complete picture, such as parental use of drug and alcohol services, community mental health services and general practice visits. Data on homelessness in the BEBOLD platform were not used as the available data did not overlap with the observation period for the study population. In addition, we were unable to assess risk factor profiles for children not born in SA. However, we do not expect children born elsewhere to have a higher risk factor profile compared to those born in SA owing to the selective nature of the Australian migration program and the state's relatively small intake (7%) [26] of total humanitarian entrants to Australia.

As this study uses linked administrative data, we are unable to examine other factors associated with CP reports, such as differences in family life and support‐seeking behaviours based on collectivist or individualist cultures, acculturation of migrant families over successive generations and migration‐related stressors [27, 28]. Similarly, we were unable to examine components of the ‘decision‐making ecology’ that may influence reporter bias, knowledge and understanding of professional obligations with regards to CP legislation [10, 13]. It is acknowledged that the composition of CALD populations reflects Australia's migration policies and global factors that contribute to immigration, such as political instability and conflict, which are associated with different health and social needs upon resettlement. As such, future analysis with updated data can assess if the patterns observed in this study are preserved.

The prevalence of risk factors for contact with CP was higher in non‐CALD than CALD children, but the opportunities to be observed for CP contact were similar. The pattern of findings persisted when children from independent and Catholic schools were included in the sensitivity analysis. CALD children have less contact with CP than non‐CALD children [1]. This is consistent with lower levels of background risk factors among CALD children, and similar contact patterns with health, education and housing systems that generate reports to CP agencies for CALD and non‐CALD children. Inclusion of general practice or primary care data to assess the opportunities for CP surveillance would be a next step in building on these findings. This is of particular importance in rural areas where primary health services are the main health care provider for the population, along with the increasing propensity of CALD migrants relocating from capital cities to regional Australia [29].

## Conflicts of Interest

The authors declare no conflicts of interest.

## Supporting information


Data S1.
