# Supplementary material for: Can Risk Factors and Opportunities to Be Observed Explain Why Culturally and Linguistically Diverse Children Have Less Child Protection Contact?
Source: J Paediatr Child Health. 2025 Mar 20;61(6):865–72. doi: 10.1111/jpc.70036 (PMC12128723; doi:10.1111/jpc.70036)
Supplement: Supplementary file 1 — Data S1. [file JPC-61-865-s001.docx]

# Supplementary

**Figure S1: Flowchart of eligible population and sample for sensitivity analysis**

Children with no mother and co-parent linked, *n = 473*

Risk factor analysis sample:

Child - mother dyad, *n = 46 104 children*

*Non-CALD: n = 41 293, CALD: n = 4811*

- Child with only mother linked, *n = 1282*
- Child with mother and co-parent linked, *n = 44 822*

Eligible: All children born in South Australia (SA) and commenced the first year of formal schooling (Reception) in all schools in AEDC years 2009, 2012 and 2015,

*N* *=* *46 577 children*

*Non-CALD: n = 41 759, CALD: n = 4818*

**Definitions**

Non-CALD: Aboriginal or Torres Strait Islander OR non-Aboriginal & English language background & child born either in English speaking country or South Africa

CALD: non- Aboriginal & LBOTE (speaks a language other than English at home &/or English is their second language) & born either in English speaking (Australia, New Zealand, United Kingdom, Ireland, United States of America, Canada)/non-English speaking country/unknown country of birth OR non-Aboriginal & English language background & born in non-English speaking country

Table S1: Health care and early childhood education system contact for children born in SA and attended reception in all SA schools in years 2009, 2012 and 2015, followed up to age 7 years

|  | **Non-CALD** (n=41 759) | | **CALD** (n=4818) | | **Total** (N=46 577) | | **Risk difference** (non-CALD - CALD) | **95% CI** |
| --- | --- | --- | --- | --- | --- | --- | --- | --- |
|  | **n** | **col %** | **n** | **col %** | **N** | **col %** | Percentage points | Percentage points |
|  |  |  |  |  |  |  |  |  |
| **Number of antenatal care visits mother attended** |  |  |  |  |  |  |  |  |
| < 7 | 2708 | 6.5 | 367 | 7.6 | 3075 | 6.6 | -1.1 | -1.8--0.3 |
| 7 to 10 | 15 725 | 37.7 | 2224 | 46.2 | 17 949 | 38.5 | -8.5 | -10.0--7.0 |
| > 10 | 20 598 | 49.3 | 1873 | 38.9 | 22 471 | 48.2 | 10.5 | 8.9-11.9 |
| Missing | 2728 | 6.5 | 354 | 7.4 | 3082 | 6.6 | -0.9 | -1.6--0.1 |
|  |  |  |  |  |  |  |  |  |
| **Completed healthcheck with CAFHS** |  |  |  |  |  |  |  |  |
| 1-4 week check | 34 184 | 81.9 | 3937 | 81.7 | 38 121 | 81.9 | 0.2 | -1.0-1.3 |
|  |  |  |  |  |  |  |  |  |
| **By age 7 years** |  |  |  |  |  |  |  |  |
| Ever presented to ED | 30 369 | 72.7 | 3949 | 82.0 | 34 318 | 73.7 | -9.2 | -10.4--8.1 |
| Ever admitted | 22 708 | 54.4 | 2641 | 54.8 | 25 349 | 54.4 | -0.4 | -1.9-1.0 |
|  |  |  |  |  |  |  |  |  |
| **Non-parental care/EC programs** |  |  |  |  |  |  |  |  |
| Yes | 38 504 | 92.2 | 4252 | 88.3 | 42 756 | 91.8 | 4.0 | 3.0-4.8 |
| No | 673 | 1.6 | 87 | 1.8 | 760 | 1.6 | -0.2 | -0.6-0.2 |
| Don't know | 2297 | 5.5 | 453 | 9.4 | 2750 | 5.9 | -3.9 | -4.8--0.3 |
| Missing | 285 | 0.7 | 26 | 0.5 | 311 | 0.7 | 0.1 | -0.02-0.4 |
|  |  |  |  |  |  |  |  |  |

Table S2: Distribution of child maltreatment risk factors for children born in SA and attended reception in all SA schools in years 2009, 2012 and 2015, followed from 12 months prior to birth up to age 7 years

|  | **Non-CALD** (n = 41 293) | | **CALD** (n = 4811) | | **Total** (N = 46 104)† | | **Risk difference**  (non-CALD - CALD) | **95% CI** |
| --- | --- | --- | --- | --- | --- | --- | --- | --- |
|  | **n** | **%** | **n** | **%** | **n** | **%** | Percentage points | Percentage points |
| **Mental health** |  |  |  |  |  |  |  |  |
| 12 months prior to child's birth | 353 | 0.9 | 33 | 0.7 | 386 | 0.9 | 0.2 | -0.1-0.4 |
| From child's birth up to age 1 year | 911 | 2.2 | 68 | 1.4 | 979 | 2.1 | 0.8 | 0.4-1.2 |
| From child's birth up to age 4 years | 3650 | 8.8 | 265 | 5.5 | 3915 | 8.5 | 3.3 | 2.6-4.0 |
| From child's birth up to age 7 years | 5176 | 12.5 | 390 | 8.1 | 5566 | 12.1 | 4.4 | 3.6-5.3 |
|  |  |  |  |  |  |  |  |  |
| **Alcohol & other drugs** |  |  |  |  |  |  |  |  |
| 12 months prior to child's birth | 81 | 0.2 | 10 | 0.2 | 91 | 0.2 | 0.0 | -0.15-0.12 |
| From child's birth up to age 1 year | 464 | 1.1 | 13 | 0.3 | 477 | 1.0 | 0.9 | 0.7-1.0 |
| From child's birth up to age 4 years | 2041 | 4.9 | 77 | 1.6 | 2118 | 4.6 | 3.3 | 2.9-3.8 |
| From child's birth up to age 7 years | 2908 | 7.0 | 126 | 2.6 | 3034 | 6.6 | 4.4 | 3.9-4.9 |
|  |  |  |  |  |  |  |  |  |
| **Intentional self-harm** |  |  |  |  |  |  |  |  |
| 12 months prior to child's birth | 15 | 0.03 | <5 | ‡ | § | 0.0 | 0.0 | -0.1-0.1 |
| From child's birth up to age 1 year | 113 | 0.3 | 7 | 0.1 | 120 | 0.3 | 0.1 | 0.0-0.2 |
| From child's birth up to age 4 years | 558 | 1.4 | 34 | 0.7 | 592 | 1.3 | 0.6 | 0.4-0.9 |
| From child's birth up to age 7 years | 861 | 2.1 | 46 | 1.0 | 907 | 2.0 | 1.1 | 0.8-1.4 |
|  |  |  |  |  |  |  |  |  |
| **Family & domestic violence** |  |  |  |  |  |  |  |  |
| 12 months prior to child's birth | 193 | 0.5 | 14 | 0.3 | 207 | 0.4 | 0.2 | 0.0-0.3 |
| From child's birth up to age 1 year | 300 | 0.7 | 29 | 0.6 | 329 | 0.7 | 0.1 | -0.1-0.4 |
| From child's birth up to age 4 years | 980 | 2.4 | 107 | 2.2 | 1087 | 2.4 | 0.1 | - 0.3-0.6 |
| From child's birth up to age 7 years | 1259 | 3.0 | 125 | 2.6 | 1384 | 3.0 | 0.5 | 0.0-0.9 |
|  |  |  |  |  |  |  |  |  |
| **Maltreatment** |  |  |  |  |  |  |  |  |
| 12 months prior to child's birth | 11 | 0.0 | <5 | ‡ | § | 0.0 | 0.0 | -0.04-0.1 |
| From child's birth up to age 1 year | 19 | 0.1 | <5 | ‡ | § | 0.1 | 0.0 | -0.1-0.05 |
| From child's birth up to age 4 years | 78 | 0.2 | 9 | 0.2 | 87 | 0.2 | 0.0 | -0.1-0.1 |
| From child's birth up to age 7 years | 108 | 0.3 | 12 | 0.3 | 120 | 0.3 | 0.0 | -0.1-0.2 |
|  |  |  |  |  |  |  |  |  |
| **Housing insufficiency** |  |  |  |  |  |  |  |  |
| 12 months prior to child's birth | 1369 | 3.3 | 224 | 4.7 | 1593 | 3.5 | -1.3 | -2.0--0.7 |
| From child's birth up to age 1 year | 2327 | 5.6 | 254 | 5.3 | 2581 | 5.6 | 0.4 | -0.3-1.0 |
| From child's birth up to age 4 years | 6602 | 16.0 | 655 | 13.6 | 7257 | 15.7 | 2.4 | 1.3-3.4 |
| From child's birth up to age 7 years | 8051 | 19.5 | 756 | 15.7 | 8807 | 19.1 | 3.8 | 2.7-4.9 |
|  |  |  |  |  |  |  |  |  |

† children born in SA linked to at least one parent

‡ Less than zero per cent

§ Redacted due to presence of cells within the same row with counts of less than five
